# The Utility of Cerebrovascular Reactivity MRI in Brain Rehabilitation: A Mechanistic Perspective

**DOI:** 10.3389/fphys.2021.642850

**Published:** 2021-03-17

**Authors:** Venkatagiri Krishnamurthy, Justin D. Sprick, Lisa C. Krishnamurthy, Jolie D. Barter, Aaminah Turabi, Ihab M. Hajjar, Joe R. Nocera

**Affiliations:** ^1^Center for Visual and Neurocognitive Rehabilitation, Atlanta VAMC, Decatur, GA, United States; ^2^Division of Geriatrics and Gerontology, Department of Medicine, Emory University, Atlanta, GA, United States; ^3^Department of Neurology, Emory University, Atlanta, GA, United States; ^4^Division of Renal Medicine, Department of Medicine, Emory University, Atlanta, GA, United States; ^5^Department of Physics & Astronomy, Georgia State University, Atlanta, GA, United States; ^6^Department of Biology, Georgia State University, Atlanta, GA, United States; ^7^Division of Physical Therapy, Department of Rehabilitation Medicine, Emory University, Atlanta, GA, United States

**Keywords:** integrative physiology, cerebrovascular reactivity, MRI, mechanism, rehabilitation, aging, cerebrovascular disease

## Abstract

Cerebrovascular control and its integration with other physiological systems play a key role in the effective maintenance of homeostasis in brain functioning. Maintenance, restoration, and promotion of such a balance are one of the paramount goals of brain rehabilitation and intervention programs. Cerebrovascular reactivity (CVR), an index of cerebrovascular reserve, plays an important role in chemo-regulation of cerebral blood flow. Improved vascular reactivity and cerebral blood flow are important factors in brain rehabilitation to facilitate desired cognitive and functional outcomes. It is widely accepted that CVR is impaired in aging, hypertension, and cerebrovascular diseases and possibly in neurodegenerative syndromes. However, a multitude of physiological factors influence CVR, and thus a comprehensive understanding of underlying mechanisms are needed. We are currently underinformed on which rehabilitation method will improve CVR, and how this information can inform on a patient’s prognosis and diagnosis. Implementation of targeted rehabilitation regimes would be the first step to elucidate whether such regimes can modulate CVR and in the process may assist in improving our understanding for the underlying vascular pathophysiology. As such, the high spatial resolution along with whole brain coverage offered by MRI has opened the door to exciting recent developments in CVR MRI. Yet, several challenges currently preclude its potential as an effective diagnostic and prognostic tool in treatment planning and guidance. Understanding these knowledge gaps will ultimately facilitate a deeper understanding for cerebrovascular physiology and its role in brain function and rehabilitation. Based on the lessons learned from our group’s past and ongoing neurorehabilitation studies, we present a systematic review of physiological mechanisms that lead to impaired CVR in aging and disease, and how CVR imaging and its further development in the context of brain rehabilitation can add value to the clinical settings.

## Introduction

The human brain requires a continuous supply of glucose and oxygen to meet continual neural metabolic demands and, therefore, relies on an intricate vascular network to maintain function. Fortuitously, several cerebrovascular mechanisms ensure sufficient delivery of glucose and oxygen to the active neural tissue, prompt removal of neural metabolic by-products, and constant global blood supply despite variations in perfusion pressure (Ozturk and Tan, 2018). This underscores the importance of vascular health and its maintenance in order to support higher order cognitive functioning and quality of life. Cerebrovascular reactivity (CVR), which reflects the ability of cerebral vessels to dilate or constrict in response to physiologic demands (Liu et al., 2019) is an important marker that plays a vital role in indexing vascular health.

Brain rehabilitation broadly has two primary components: (i) relearning lost or forgotten skills and (ii) compensating for more enduring impairments. After an injury or degeneration, the brain spontaneously begins to heal *via* plastic changes, which may include the repair of neural and vascular residual tissue and rewiring or remodeling for healthy tissue to assume the function of lost/degenerating tissue (by generating new neural and axonal pathways and blood vessels). While spontaneous recovery is extremely important, evidence-based clinical outcomes suggest that most patients will need specialized rehabilitation programs to achieve successful long term cognitive and functional outcomes. Thus, from the brain rehabilitation standpoint, one can imagine the paramount significance of characterizing and quantifying vascular health, which highlights the clinical importance of CVR measurement.

Neuroimaging is a rising, prominent, and clinically attractive technique that can play a significant role in neurorehabilitation – particularly, to measure and predict brain plasticity and reorganization in response to therapy (Crosson et al., 2017, 2019). CVR acquired using MRI is a relatively nascent and promising technique that has not yet made sufficient inroads into neurorehabilitation. The goal of this review is to critically present some of the challenges that currently preclude CVR MRI’s potential as an effective diagnostic and prognostic tool in treatment planning and guidance. Further, some widely used rehabilitation approaches such as aerobic exercise naturally invoke cardiovascular responses that dynamically interplay with cerebrovascular function. Thus, cerebrovascular control and its integration with other physiological systems (such as cardiovascular and pulmonary) play a key role in effectively describing CVR’s role in brain rehabilitation. To our best knowledge, we believe that the existing CVR imaging literature describing underlying mechanisms is incomplete as the scope is perhaps limited to just the brain. First, we describe the mechanisms underlying CVR in the context of rehabilitation science. Second, we discuss CVR impairment in aging, aging-related neurodegenerative, and cerebrovascular diseases as well as multi system level pathology (e.g., renal disease) employing an integrative physiology approach to promote a holistic understanding of cerebrovascular physiology and its role in brain function. Finally, based on the lessons learned from our group’s past and ongoing neurorehabilitation studies, we formulate cutting-edge ideas for how CVR imaging can add value to clinical brain rehabilitation settings.

## Mechanistic Outlook

### Cerebrovascular Response to a Bolus of CO_2_

Cerebrovascular reactivity refers to the ability of cerebral vessels to constrict, and (more commonly) to dilate in response to changes in arterial pressure of CO_2_ (PaCO_2_). The site of regulation has historically been attributed to the arterioles (Lennox and Gibbs, 1932) because the greatest pressure drop along the vascular tree occurs there. Although arterioles do play a major role in regulation of cerebral blood flow, larger vessels also contribute to resting cerebrovascular tone (Faraci and Heistad, 1990) and mediate CVR. Specifically, extracranial vessels in the neck [e.g., internal carotid artery (ICA); Willie et al., 2012; Hoiland et al., 2016] and larger intracranial vessels [e.g., middle cerebral artery (MCA); Coverdale et al., 2014, 2017; Verbree et al., 2014] also respond to changes in PaCO_2_ and contribute to the CVR response. The role of large arteries in mediating CVR is important from a clinical and rehabilitative standpoint because it suggests that disease processes that affect large arteries such as atherosclerosis may in turn negatively affect CVR. Additionally, regional differences within the cerebral circulation are also present, with the anterior circulation exhibiting a greater CVR relative to the posterior circulation (Willie et al., 2012; Hoiland et al., 2019).

### Molecular Signaling Mechanisms of CVR

**Figure 1:**
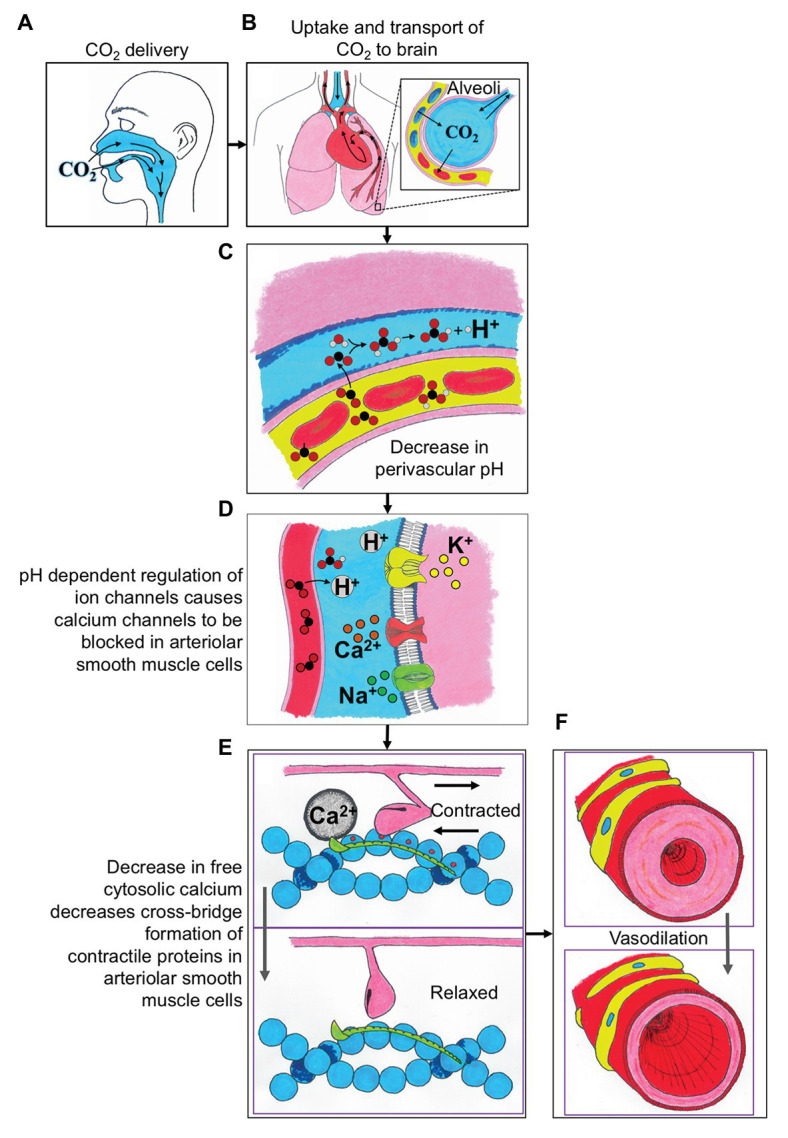
A schematic describing the integrative physiologic mechanisms governing cerebrovascular reactivity (CVR) in response to elevated carbon dioxide (CO_2_) stimuli. **(A)** The CO_2_ is delivered to the participant *via* mouth or nose. **(B)** The uptake of CO_2_ is in the alveoli of the lungs, where the gas is exchanged into the blood, and then transported to the brain. **(C)** The CO_2_ exchanges from the blood into the perivascular space and causes a decrease in perivascular pH. **(D)** The decreased perivascular pH causes the calcium channels to be blocked in arteriolar smooth muscle cells. **(E)** Decreased local calcium concentration results in relaxation of arteriolar smooth muscle cells. **(F)** The relaxation of arteriolar smooth muscle cells leads to local vasodilation and subsequent CVR contrast. H^+^, proton; Ca^2+^, calcium; Na^+^, sodium; and K^+^, potassium.

**Figure 2:**
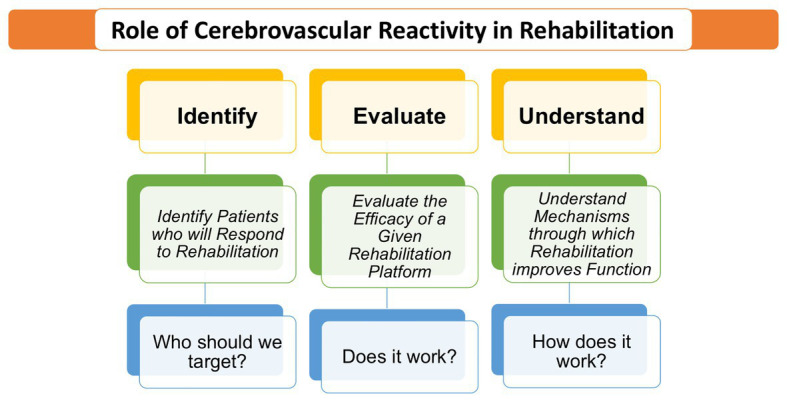
A graphical flowchart depicting the utility of CVR imaging in rehabilitation.

An important relationship that should be clarified is that while carbon dioxide (CO_2_) is the delivered stimulus during the majority of CVR assessments, CO_2_ is tightly coupled to pH, and increases in CO_2_ are accompanied by reductions in perivascular pH (Figure 1C). As comprehensively discussed by Hoiland et al. (2019), it is this reduction in perivascular pH that mediates changes in cerebrovascular tone and serves as the basis for CVR assessment, and hence reflecting chemo-regulation of the cerebral perfusion. The mechanisms underlying CVR can further be organized into two components: (1) signal transduction: the ability of cerebral vessels to detect changes in PaCO_2_/perivascular pH (Figures 1A–C) and (2) vasomotor response: the ability of cerebral vessels to dilate or constrict in response to these changes (Figures 1D–F). Signal transduction of CVR is accomplished *via* changes in resting membrane potential and is regulated by membrane-bound ion channels on vascular smooth muscle cells. Numerous channels have been implicated in the signal transduction of CVR, including calcium-activated potassium channels (KCa channels), voltage-gated potassium channels (Kv channels), and ATP-sensitive potassium channels (KATP channels), and the reader is referred to a comprehensive review for an in-depth discussion on this topic elsewhere (Kitazono et al., 1995). CVR thus represents a complex phenomenon that involves multiple steps of regulation, including sensing changes in perivascular pH, modification of intracellular free calcium, and activation of the contractile machinery (Figure 1).

### Interaction of CVR With Other Cerebrovascular Control Mechanisms

While CVR is undoubtedly a useful biomarker for cerebrovascular responsiveness, it represents only one of several key mechanisms underlying cerebral blood flow regulation. Other factors known to influence cerebrovascular tone include the chemoreflex mediated changes in sympathetic nerve activity which subsequently drive increases in arterial pressure, cerebral autoregulation (the mechanism through with the cerebral vasculature buffers these changes in arterial pressure), and neurovascular coupling (Willie et al., 2014). Since all of these factors can independently modulate CVR, their interaction must be considered when evaluating CVR and/or assessing change brought on by rehabilitation.

Chemoreflex activation promotes increases in sympathetic nerve activity in response to a bolus of CO_2_ (Jouett et al., 2015), and this in turn increases mean arterial pressure (MAP; Coverdale et al., 2017). Since increases in MAP drive increases in cerebral perfusion, it is important to account for this response when interpreting CVR data (Regan et al., 2014; Hoiland et al., 2019). Coverdale et al. (2017) compared CVR and MAP during a 6% CO_2_ challenge in young (age = 24 ± 4 y.o.) and older (age = 66 ± 7 y.o.) adults and normalized cerebrovascular conductance (the quotient of cerebral blood flow and MAP) to gray matter volume in the M1 segment of the MCA territory. Overall, CVR was similar between groups; however, the older adults exhibited a greater increase in MAP and an attenuated cerebrovascular conductance compared to their younger counterparts. Thus, the role of cerebral hemodynamics may play a greater role in mediating CVR in older adults due to increased MAP in response to CO_2_. When considering how CVR is altered in aging and aging-related disease, or improved following rehabilitation, it is essential that the effects of chemo-reflex mediated increases in blood pressure, and subsequent changes in cerebral hemodynamics (including CVR) at whole brain level ought to be considered.

Cerebrovascular reactivity is influenced by arterial pressure through its interaction with cerebral autoregulation. Cerebral autoregulation is the mechanism through which cerebral vessels regulate blood flow over a wide range of arterial pressures. Severe hypotension dampens CVR (Harper and Glass, 1965), while autoregulation is attenuated during hypercapnia (Aaslid et al., 1989; Maggio et al., 2013; Perry et al., 2014). This interaction stems from the fact that both of these mechanisms rely on the same vascular reserve and simultaneously work to maintain cerebral perfusion. In other words, there is a physical limit to maximal vessel diameter, and whether increases in vessel diameter are mediated through reductions in MAP, autoregulation, or through increases in PaCO_2_ (CVR) will depend on the sensitivity of these two mechanisms individually, as well as their interaction. It was recently shown that impairments in CVR correlate with impairments in cerebral autoregulation (Klein et al., 2020), suggesting that dysregulation of both mechanisms stems from a common signaling pathway. It is currently unknown if this commonality is due to a dysregulation of signal transduction (e.g., sensing changes in PaCO_2_ or MAP) or if structural abnormalities in the vascular wall (e.g., atherosclerosis or calcification) are responsible. It is conceivable that a calcified vessel may be less responsive to all vasoactive stimuli in general and may, therefore, influence both cerebral autoregulation and CVR.

Interactions between CVR, arterial pressure/cerebral autoregulation, and neurovascular coupling are important from a rehabilitative standpoint because these mechanisms may exhibit different responses to a given rehabilitation platform. Maggio et al. (2013) explored the interaction between neurovascular coupling and CVR by measuring changes in cerebral blood flow in response to passive arm movement during normo- and hypocapnia and found that neurovascular coupling was impaired during the hypercapnia trial. Similarly, Szabo et al. (2011) also observed impairments in neurovascular coupling, as measured through visual stimulation, during hypocapnia induced *via* hyperventilation. Although speculative, it is possible that assessing multiple cerebrovascular control mechanisms together may provide additional mechanistic insight that cannot be captured by a single measurement alone. For example, improvements in more than a single mechanism following rehabilitation may reflect improvements in overall vascular compliance since the mechanisms depend on the ability of cerebral vessels to dilate in response to a given stimulus. Conversely, improvements in a single regulatory mechanism (i.e., CVR, cerebral autoregulation, MAP, or neurovascular coupling) may provide diagnostic capability of which regulatory component is improving due to rehabilitation while the remainder continues to be impaired. Taken together, it is clear that measuring multiple indices of cerebrovascular function in addition to CVR, may provide a more granulated understanding of the benefits and limitations of a given rehabilitation platform, and potentially how to tailor such rehabilitation platforms to improve specific cerebrovascular impairments.

### Mechanisms Leading to CVR Impairment

#### Arterial Stiffness

Arterial stiffness refers to the gradual stiffening of the arterial wall and is indicative of reduced vascular compliance. While some degree of stiffening is to be expected in aging, research over the last two decades has highlighted the role of arterial stiffness in the pathogenesis of cardiovascular and neurovascular disease. Importantly, increase in arterial stiffness is associated with future risk of cardio and neurovascular disease (Mitchell et al., 2010; Tsao et al., 2016). The mechanisms underlying the pathogenesis of arterial stiffening are complex and related to a multitude of inflammatory, dietary and lifestyle choices, and hemodynamic changes (Zieman et al., 2005). The gold-standard approach to assess arterial stiffness is to measure the carotid-femoral pulse wave velocity (Laurent et al., 2006), now commonly used in many research settings to investigate the link between arterial stiffness and cerebrovascular health.

Arterial stiffness may be associated with cerebrovascular dysfunction due to loss of vessel elasticity in compliant vessels. For example, vessel elasticity promotes the Windkessel effect, where some blood to be stored within the vessel during diastole (Belz, 1995), resulting in a buffering of pulsatile flow and maintenance of blood pressure during the cardiac cycle. When this buffering capacity is reduced due to arterial stiffening, transmission of increased pressure swings to the microcirculation is impacted. Cerebral vessels are particularly vulnerable to this increased pulsatile stress because the cerebrovascular tree contains relatively short arterioles (Ito et al., 2009). Thus, long term increases in pulsatile flow with arterial stiffness are thought to contribute to microvascular damage within the brain (O’Rourke and Safar, 2005).

#### Endothelial Dysfunction

Endothelial dysfunction is a hallmark of vascular disease (Rajendran et al., 2013) and is associated with reduced nitric oxide (NO) bioavailability (Hadi et al., 2005). This decreased bioavailability may also affect CVR since NO plays a key role in modifying CVR. Dietary and pharmacologic strategies that improve NO bioavailability have been shown to improve CVR. Seven days of dietary nitrate supplementation (an NO donor) improves CVR in young adults in a sex-specific manner (Fan et al., 2019). In a similar vein, statins have also improved CVR (Sander et al., 2005), likely through a NO-dependent mechanism (Yamada et al., 2000). These studies suggest that the vascular endothelium may be one viable target for rehabilitation platforms. Indeed, improvements in peripheral endothelial function are commonly assessed following rehabilitation (Cornelissen et al., 2014; Gelinas et al., 2017; Khorvash et al., 2020). The most common of which is perhaps flow-mediated dilation (FMD). This biomarker has powerful prognostic value, since a 1% improvement in FMD is associated with a 13% risk-reduction of future cardiovascular disease (Inaba et al., 2010). Interestingly, however, peripheral measures of endothelial function do not always correlate with CVR (Palazzo et al., 2013; Carr et al., 2020; Junejo et al., 2020). This suggests that brain specific methodologies to assess endothelial function (i.e., CVR) may be a more appropriate outcome for brain rehabilitation platforms.

## CVR MRI and ITS Utility in Brain Rehabilitation

### CVR as Dignaotic and Prognostic Biomarkers

With the mechanistic outlook described above, we now turn our attention to the utility of CVR in rehabilitation. Rehabilitation can be thought of in the realm of “biomarkers” wherein broadly, clinicians are interested to detect degeneration, disease processes, and assess treatment success in rehabilitation. Such an approach with vascular imaging techniques has been relatively less undertaken due to technological limitations and/or difficulty with clinically translatability. However, the importance of evaluating cerebral vascular health and response to rehabilitation cannot be overstated. Longitudinal studies show a positively correlated relationship between midlife hypertension (around 50 years old) and cognitive decline (20–25 years later) or dementia (Alzheimer’s or vascular) at more advanced ages (Duron and Hanon, 2008). Similarly, aging and hypertension are major risk factors for ischemic stroke (Shekhar et al., 2017). Together, these age-related pathologies are associated with degradation of vascular smooth muscle, endothelial cells, disruption of chemoreceptor sensitivity as well as MAP, autoregulation, and neurovascular coupling. Given the integration of these markers on CVR and the implications for rehabilitation, there is an immense need to continue to develop non-invasive vascular biomarkers such as CVR. This will assist rehabilitationists and clinicians with more accurate diagnoses and treatment planning. Further, an improved understanding of mechanistic underpinnings demonstrated in the successes of rehabilitation will allow for improved individualized outcomes (see Figure 2). With the highlighted importance of CVR as a tool in rehabilitation, we now describe CVR as biomarkers that can aide to identify/detect vascular dysfunction and as prognostic biomarkers that are helpful in evaluating prediction of treatment efficacy.

With cutting edge technology, CVR is well poised to generate clinically relevant, innovative biomarkers that are sensitive and can determine the vascular territory that is deficient, providing essential information to guide rehabilitation planning. In the adaptive stage (prior to disease onset), decline in some physical properties (such as aging-related increase arterial stiffness) do not yet create a pathological change but elicit an adaptive shift in some physiologic properties (such as an increase in MAP). Detection of cerebrovascular changes at this stage is beneficial to engage in disease prevention measures (e.g., a prescribed exercise regimen to limit risk factors associated with stroke and vascular dementia, for example). Next, in the early pathology stage (at disease onset), the body has spontaneous or natural adaptive mechanisms that can repair damage to restore function. In this stage, measurement of CVR may be helpful to identify patients who will respond to a particular form of rehabilitation. For example, in a stroke model, CVR can be beneficial at this stage to identify brain areas that are undergoing spontaneous vascular repair, and brain areas that need further treatment to promote such repair (Gabriel-Salazar et al., 2018). This is the stage in which prognostic biomarkers can be most powerful because they can provide a treatment window to repair damage and restore function. Subacute phase of stroke, early stages of dementia (Holmes et al., 2020), and mild cognitive impairment (MCI) are a few sample scenarios that fit the need for diagnostic CVR-based biomarkers that can be helpful in evaluating the effectiveness of a specific treatment. In the late pathology stage (i.e., chronic conditions), the disease may result in irreversible damage [for example, later stages of Alzheimer’s disease (AD)]. CVR based biomarkers at this stage are still useful for detection and characterizing the extent of the disease to help design treatments to slow or halt progression of the disease. To summarize, in order to assess the effectiveness of rehabilitation, CVR-based biomarkers are vital to help identify brain areas that are responsive and/or non-responsive to initial repair, and to quantify the response over the course of disease progression and its treatment.

During the chronologic phases of disease evolution and treatment-induced recovery, prediction of such processes can be immensely helpful to clinical care. By understanding the biological processes involved in cerebrovascular rehabilitation, CVR-based predictive biomarkers promises to catapult rehabilitation forward to provide personalized and patient centric care *via* more accurate patient stratification (i.e., identify and evaluate components of Figure 2) and resulting in increased effective outcomes during recovery. An example of how CVR can improve existing imaging technologies to have better prognostic value, CVR can be combined with task- or resting state-functional MRI (fMRI) to enhance the neuro-sensitivity. The blood oxygen level dependent (BOLD) fMRI is a widely used approach to index neural plasticity in rehabilitation neuroimaging (Crosson et al., 2017). However, it is known that BOLD fMRI signals can be reduced without the presence of a functional deficit (Krainik et al., 2005), potentially leading to misinterpretations of fMRI activations maps in disease and limiting the applicability of this imaging in rehabilitation. This is because BOLD fMRI is a complex neurovascular signal, wherein baseline vascular variability could mislead the interpretation of longitudinal rehabilitation-induced neural plasticity. From this standpoint, some studies (Bandettini and Wong, 1997; Liu et al., 2013) have shown that CVR maps can be retrospectively combined with BOLD fMRI data to sensitize BOLD to more accurately index neural activity. Thus, in the context of diagnostic and prognostic biomarkers for neurocognitive rehabilitation, CVR maps might be extremely helpful to enhance the accuracy of widely employed BOLD maps and its interpretation.

### Standardization of CVR Measures

Given that rehabilitation involves longitudinal measures to either monitor brain plasticity and remodeling or predict brain changes over a period of time, it is important to standardize desired rehabilitation biomarkers (i.e., CVR in our case). Without a standard approach, different CVR methods with non-reproducible stimuli can result in reduced test re-test reliability of CVR outcomes (Fierstra et al., 2013; Fisher, 2016), thereby reducing the sensitivity to rehabilitation-related changes. In fact, a detailed comparison of practical concerns such as complexity/simplicity, effectiveness and cost between the fixed inspired concentration methods and computer-controlled prospective targeting is well-described in (Fisher, 2016), wherein the author makes a strong case for how standardized and repeatable stimuli are more reliably delivered *via* the computer-controlled systems.

In addition to standardization of stimulus delivery, objective transformation of voxel-wise CVR maps is necessary for more accurate clinical interpretation of CVR because the range of CVR can vary due to factors such as age, gender, and regional variability in both healthy and even more pronounced in diseased populations. In fact, (Sobczyk et al., 2015) have put forth an elegant approach that demonstrates that *z*-map scoring and analysis of CVR studies have the potential to reduce confounding effects of test-to-test, subject-to-subject, and platform-to-platform variability for comparison of CVR, and thereby enhancing objective evaluation of abnormality, and to characterize the extent and distribution of pathophysiology in patients with cerebral vasculopathy. Further, given that individualized treatment planning is highly preferred in areas such as stroke rehabilitation, there is an immense need for standardized approaches to evaluate the within subject change in measured CVR relative to the expected reproducibility of the test. Recent methodological development in this line of thought (Sobczyk et al., 2016) facilitates conceptualizing experiments to test if variations in CVR outside the range of these normal test-to-test changes are attributable to pathophysiologic changes.

## CVR with an Exemplar Rehab Platform in Aging and Aging Related Diseases

### Aging and Exercise

Aging-related pathology and physiological changes in cerebrovascular function are well-documented (Lu et al., 2011) and recent epidemiological data indicate considerable overlap between cerebrovascular dysfunction and neurologic pathology (Toledo et al., 2013; Attems and Jellinger, 2014). Importantly time spent in a “sub-clinical” (adaptive) stage appears to be years or even decades before crossing a diagnostic threshold of overt neurocognitive and behavioral pathology (Sperling et al., 2014) and spans a significant amount of time to initiate targeted interventions for limiting cerebrovascular dysfunction. It is increasingly documented that rehabilitation, and specifically exercise, can slow the progression of cerebrovascular pathology in older adults. In fact, it has been demonstrated that of all modifiable risk factors for vascular related pathology, decreasing sedentary behavior is the most statistically significant and effective measure to counter cerebrovascular dysfunction and associated cognitive decline in adults.

Exercise can improve overall brain health and function (see the seminal review of the effects of fitness on cognition by Colcombe and Kramer (2003). Exercise has repeatedly improved performance for psychomotor and processing speed, working memory, inhibition, verbal fluency, and spatial memory (Erickson and Kramer, 2009; Wasylyshyn et al., 2011; Holzschneider et al., 2012; McGregor et al., 2013; Nocera et al., 2015). However, the mechanistic underpinnings of these improvements have been elusive. Additional research has documented that older adults with high aerobic capacity produced activity patterns in dorsolateral prefrontal areas similar to those of young adults during an executive function task (Colcombe and Kramer, 2003), motor control (McGregor et al., 2011), category member generation (Nocera et al., 2017), semantic memory (Smith et al., 2011), and inhibition tasks (Prakash et al., 2011). These studies strongly implicate physical activity as a rehabilitative platform to alter, maintain, and improve the health of the central nervous system; however, what is less known, is the role of how the cerebral vasculature changes in response to exercise.

Much of the groundwork laid for the impetus of exercise beneficially impacting cerebrovascular function has been based on findings in the peripheral vasculature. Aerobic exercise is associated with a multitude of cardiovascular benefits, including improvements in arterial compliance and endothelial function (Santos-Parker et al., 2014). Arterial compliance is related to vascular smooth muscle tone and the structure within the arterial wall and extracellular matrix (Seals et al., 2014). Thus, aerobic exercise prevents arterial stiffness and reverses any stiffening that may occur with age or pathology. Similarly, repeated studies demonstrate that aerobic exercise protects against the development of endothelial dysfunction (Walker et al., 2009, 2014). These studies, in both young and older aerobically-trained adults demonstrate greater NO bioavailability as the primary driver of a healthier endothelial profile. The mechanistic basis for this is thought to be an attenuation of age-related reduction in endothelial NO synthase (Tanabe et al., 2003), and an improved redox balance resulting in less NO scavenging.

While it is appealing to suppose that improvements in peripheral vascular function extend to the cerebral circulation, peripheral and cerebrovascular indices of vascular function do not always track. This discrepancy necessitates the need for cerebral-specific markers of vascular function, one of which is CVR. Nonetheless, these highlighted studies of peripheral vasculature and early studies examining the cortical flow provide evidence that cerebrovascular targets may also be adaptable to rehabilitation, but this has been generally less explored in humans. A cross-sectional study by Ainslie et al. (2008) demonstrated that middle cerebral artery blood velocity (MCAv) across the adult lifespan increased 10–25% with greater aerobic fitness. This finding was substantiated in a longitudinally study wherein an 8-week aerobic exercise intervention improved regional CBF in sedentary older men aged 60–70 (Kleinloog et al., 2019).

Although exercise training may attenuate age-related reductions in cerebral blood flow, the role of exercise training in CVR is more complex. Murrell et al. (2013) reported elevated CVR at rest and during exercise following a 12-week exercise intervention, irrespective of age, while Miller et al. (2018) observed no differences in CVR between habitually active young and older adults. Thomas et al. (2013) observed an attenuated CVR in 10 masters athletes (74.5 ± 5.8 y.o.) in comparison to 10 sedentary elderly individuals (75.4 ± 5.6 y.o.) suggesting that CVR may be affected by lifelong exercise. One explanation for this rather counterintuitive finding may be that frequent bouts of hypercapnia *via* regular exercise over a lifetime desensitize cerebral arteries, in which case the reduced CVR may not actually reflect a lower vascular compliance *per se*, but rather attenuation in the signal.

One recent study by Intzandt et al. (2020) did use a cross-sectional approach to relate aerobic capacity (VO_2max_) to CVR in a cohort of elderly adults (55–72 years). Interestingly, these investigators observed a negative association between aerobic fitness and CVR in which individuals with reduced levels of fitness actually exhibited higher CVR. This finding, although surprising, is consistent with the study by Thomas et al. (2013). Clearly, the relationship between exercise training and CVR is complex, and the use of MRI-based CVR mapping will allow for further understanding of the interplay between exercise training, aerobic fitness, cerebrovascular dynamics, and cognitive function, but may require a multimodal approach.

To date, there are few longitudinal training studies using MRI to measure CVR following exercise training. However, research is supportive of the role of rehabilitation and exercise to promote overall cerebrovascular health. Regular exercise promotes angiogenesis, neurogenesis, and synaptic plasticity (Isaacs et al., 1992; Churchill et al., 2002; Vaynman and Gomez-Pinilla, 2006) which are markers of improved cerebral vascular function. Additionally, exercise and higher cardiorespiratory fitness have been positively linked with improved flow *via* improved resistance and compliance (Bailey et al., 2013; Zimmerman et al., 2014). Future works aimed to aid our understanding of how the cerebrovasculature responds to exercise and improved cardiovascular fitness in older adults susceptible to cerebrovascular dysfunction needs further exploration. Further, various exercise approaches [strength training, steady state aerobic, and high intensity interval training (HIIT), for example] should continue to be investigated. In addition, dosage and intensity questions remain. Lastly, biomarkers that can identify those who might differentially benefit from the various rehabilitation approaches need to be explored.

### Stroke and Non-invasive Brain Stimulation

Each year, there is approximately 795,000 stroke cases recorded in the United States (Shekhar et al., 2017), and the annual cost to treat stroke survivors is approximately 40 billion dollars in the United States and is projected to rise (Skolarus et al., 2016). Given that stroke has vascular etiology, there is an immense need to add sensitive and reliable vascular biomarkers such as CVR to improve patient care and reduce unnecessary treatment costs.

The established outlook of stroke-related neurovascular repair has changed in the last decades in favor of a more global understanding of the brain as a whole and now includes other cell types (e.g., glia and inflammatory and progenitor cells), the extracellular matrix, and the communication between these components. Several of these endogenous mechanisms such as angio-vasculogenesis (the formation of new blood vessels) are activated in the minutes following the ischemic trigger in the peri-cavitational and peri-lesional brain areas (Esquiva et al., 2018). Further, angiogenesis and vascular remodeling are found to remain elevated for days to several weeks as a response to increased collateral blood supply that is tightly coupled to neurogenesis and oligodendrogenesis as part of the endogenous neural repair (Gabriel-Salazar et al., 2018). Thus, acquiring CVR maps during the acute and early subacute stages of stroke recovery will help clinicians in identifying the rehabilitation potential of vascular repair to design effective personalized treatments. Vascular compromise of major arteries results in reduced CVR in downstream vessels, and reports show that CVR does not change from sub-acute to chronic stages (Geranmayeh et al., 2015), but remains lower compared to healthy controls during the chronic stage (Zhao et al., 2009). Specifically, the trajectory of change in CVR from acute to chronic stages, and how it predicts functional and cognitive recovery particularly during the critical sub-acute time window is an understudied research area that is currently ongoing in our studies.

In terms of rehabilitation platforms tailored to stroke, non-invasive brain stimulation techniques such as transcranial direct current stimulation (tDCS) is widely used to induce neural plasticity and brain reorganization (Crosson et al., 2017, 2019). Although tDCS is known to induce and promote neural plasticity to improve functional and behavioral gains (Crosson et al., 2019), recent reports also indicate that tDCS is associated with stimulation of endothelial cells, thereby increasing the vascular endothelial growth factor (VEGF) production that may modulate angiogenesis (Zhao et al., 2012). Further, tDCS-induced changes in pericyte membrane potential may hyperpolarize the cell and induce vasodilatory signals *via* increased prostaglandin E2 which in turn will induce capillaries vasodilatation by activating K^+^ currents in pericytes (Hall et al., 2014). Given the utility of neuroimaging (particularly CVR in this case) to measure and predict regional vascular reserve, from a rehabilitation perspective, developing and optimizing sensitive vascular measures to accurately predict behavioral recovery is necessary to improve treatment planning in stroke patients.

### MCI, Vascular Dementia, and Pharmacological Intervention

Cerebrovascular dysfunction can contribute to the manifestation of MCI and increase the risk of dementia [i.e., AD or vascular dementia (VaD)]. MCI is a state of cognitive decline, but people are still able to perform activities of daily living. VaD is the second most common form of dementia after AD and results from cerebrovascular abnormalities including lacunar infarcts, small vessel disease, and chronic cardiovascular disease (Grinberg and Heinsen, 2010). The two key components of cerebral hemodynamic control, autoregulation, and chemoregulation, may be altered in AD (Iadecola, 2004; Hajjar et al., 2014; Marmarelis et al., 2014b). It is unclear if this is an early vs. late manifestation of cerebrovascular dysfunction. Recent focus is on studying the prodromal state of MCI. The major limitation of prior research has been related to technical and methodological constraints. Nearly all our knowledge about changes in autoregulation and CVR in AD are derived from TCD studies, which can only assess blood flow velocity in the middle cerebral artery (Marmarelis et al., 2014a,b, 2017). Such an approach typically requires specialized laboratory with abilities to measure CA and CVR using beat-to-beat observations of mean arterial blood pressure (MABP), cerebral blood flow (CBFv), and end-tidal CO_2_ (ETCO_2_). The result is a description of the dynamic (time/frequency dependent) CA and CVR. Prior evidence also suggests that there is variable pattern of increased/decreased perfusion in AD brains (Dai et al., 2009).

While the underlying mechanisms for how the changes in vascular function may lead to poor cognition are debated, one possible mechanism is hypoperfusion (Moretti et al., 2008; Qiu et al., 2009; Rose et al., 2010). Aging and hypertension are associated with cerebral-microvascular disease which can lead to regional hypoperfusion and cognitive decline, including MCI and VaD (de Groot et al., 2001; Vermeer et al., 2003; Waldstein et al., 2005, 2008; Ikram et al., 2006; Manschot et al., 2006). Meta-analysis studies have highlighted the importance of vascular risk factors, including hypertension, stroke, and obesity, at the onset and progression of dementia (Savva et al., 2010; Anstey et al., 2011; Sharp et al., 2011). Due to the high prevalence (65–75%) of hypertension in older adults, many studies have addressed the relationship between blood pressure (BP) and cognitive dysfunction (Qiu et al., 2005; Novak and Hajjar, 2010). Hypertension significantly increases the risk of MCI by 1.4 times (Sierra et al., 2012), and vascular dementia by 1.5 times (Sharp et al., 2011). While aging is a significant risk factor, longitudinal studies have found that hypertension in middle age increases the risk of developing cognitive impairments and dementia later in life (Kilander et al., 2000; Kivipelto et al., 2001; Whitmer et al., 2005) highlighting the importance of regulating BP at middle age to hopefully prevent cognitive decline later in life.

Cerebrovascular reactivity is a potential biomarker for monitoring the relationship between perfusion and cognition. Previous research has established a slower CVR response to CO_2_ in individuals with MCI and AD compared to healthy older adults (Glodzik et al., 2013; Richiardi et al., 2015). Further, CVR correlates with poorer cognitive function. These changes in CVR can alter neurovascular coupling, identifying a possible underlying mechanism by which vascular function alters cognition (Novak and Hajjar, 2010). Additionally, because hemodynamic disturbance in CBF is common in both AD and VaD, studies have investigated the different patterns of regional cerebral blood flow (rCBF) and CVR in these two types of dementia. Results showed that, rCBF was significantly lower in both bilateral frontal and temporal lobes in the AD group and lower in left frontal and temporal white matter in patients with VaD. CVR calculated by rCBF was impaired more severely in bilateral frontal cortices in AD. Conversely, transcranial Doppler (TCD) tests failed to demonstrate significant difference in mean flow velocity and CVR between the two groups (Gao et al., 2013). It is concluded that the different patterns detected by ASL in resting rCBF differences and CVR in response to carbogen inhalation may serve as a potential marker to distinguish AD and VaD. Low CVR seems to be detectable even in the absence of cognitive symptoms but in those at high risk for AD, e.g., those with APOe4 carriers. In 625 adults (mean age = 78, 65% women, 22% Black) enrolled in the MOBILIZE Boston study (Leveille et al., 2008), the APOE4 genotype was associated with lower CVR (*p* = 0.04) and only APOE4 carriers with low CO_2_-reactivity had slower performance on trail making test, a measure of executive function (*p* = 0.036) and Hopkins verbal Learning test, a measure of episodic memory (*p* = 0.04; Hajjar et al., 2015).

Clinical trials and meta-analyses summarizing the longitudinal use of antihypertensives to alter progression of dementia have demonstrated conflicting results. While some studies have reported no relationship between high blood pressure and dementia (Morris et al., 2001; Lindsay et al., 2002), more studies indicate that antihypertensives can protect against cognitive impairment (Cacciatore et al., 1997; Murray et al., 2002; Hajjar et al., 2005) and reduce the risk of developing dementia, specifically VaD (In’t Veld et al., 2001; Qiu et al., 2003; Yasar et al., 2005). Further, the class of antihypertensive medication may enhance the beneficial effects on cognition. A previous study demonstrated that individuals with MCI that took a renin-angiotensin system (RAS)-acting antihypertensive medicine for at least 3 years were less likely to develop AD (Wharton et al., 2015). Specifically, RAS-acting antihypertensives that cross the blood brain barrier (BBB) conferred greater cognitive benefits. Very few studies have addressed the relationship between antihypertensives, dementia, and CVR. One study reports that angiotensin receptor blockers may preserve cerebral hemodynamics (i.e., cerebral blood flow and CVR) and executive function (Hajjar et al., 2013). However, this study was not performed in individuals with dementia. Therefore, studies are needed to address if antihypertensives reduce the risk of MCI and dementia by improving cerebrovascular function.

### Renal Disease and Exercise

Additional populations that may benefit from CVR assessments are patients with end-stage kidney disease (ESKD). These patients experience reductions in cerebral blood flow during hemodialysis, and this is associated with cognitive dysfunction (Findlay et al., 2019). Given the high frequency of hemodialysis administration (typically three to four times a week), there is a growing need for therapeutic strategies to attenuate or prevent intradialytic reductions in cerebral oxygen delivery. Intradialytic declines in cerebral perfusion may be partially opposed by increases in PaCO_2_ resulting from bicarbonate shifts during the dialysis treatment (Sprick et al., 2020). Thus, improving CVR with rehabilitation may be one strategy to attenuate the incidence and severity of brain injury secondary to hemodialysis. One rehabilitation platform that could potentially be utilized for this purpose is intradialytic cycling exercise. A recent pilot study demonstrated that 3 months of intradialytic cycling was able to preserve cognition in ESKD patients; however, the mechanism underlying this improvement remains to be clarified (McAdams-DeMarco et al., 2018). Although speculative, one possibility is that acute increases in PaCO_2_ that occur with exercise (Moraine et al., 1993) promote cerebrovascular dilation *via* CVR. Future work should clarify the role of CVR in opposing intradialytic reductions in cerebral blood flow, and how other rehabilitation platforms may be adapted to the dialysis setting to target CVR.

## Rehabilitation Relevant Advanced Imaging Approaches Involving CVR

In light of the theme for this special topic which is CO_2_-based CVR measures, we have consistently focused on CO_2_-induced vasodilation mechanisms and its utilities in rehabilitation. Indeed, CO_2_-based CVR approach inherently has a high sensitivity and robustness in indexing CVR. However, its utility in various rehabilitation settings is currently limited due the need for CO_2_ gas inhalation, time consuming setup of the apparatus, and burden on patient’s comfort. To overcome some of these clinically translatable issues, resting-state functional MRI (rsfMRI) scans to derive CVR maps have been explored (Golestani et al., 2016; Liu et al., 2017). Such an approach is practically clever as it is not only compatible with clinical use due to limited patient burden, but also the versatility of rsfMRI allows a “one-stop shop” platform wherein multiple clinically relevant functional and physiological metrics can be derived from a single rsfMRI scan. Specific to CVR, cued breathing during the rsfMRI scan actively engages the participant to enhance fluctuations in their ETCO_2_ level to modulate their breathing (Liu et al., 2020). However, while rsfMRI based CVR is quite attractive, subjects who have small fluctuations in their natural breathing pattern may not be ideal candidates because the derived CVR metrics may be noisy and unreliable for longitudinal rehabilitation assessments. One caveat with the intermittent modulation approach is that cued breathing can invoke meditative brain states (Park and Park, 2012) and/or mimic respiratory vagal nerve stimulation relegating autonomic responses as the driver of cued breath modulations (Gerritsen and Band, 2018). Thus, the intermittent modulation approach can interfere or dilute the effects of cognitive rehabilitation and adjuvant therapy-based strategies. In sum, these proof of principle advanced imaging approaches to derive clinically-friendly CVR maps are promising, but will need to be tailored and extensively developed for the rehabilitation setting.

Cerebrovascular reactivity can be retrospectively combined with task fMRI data to sensitize the BOLD signals to index neural activity (Bandettini and Wong, 1997; Liu et al., 2013). Given that profiling and characterizing behavioral changes is important for cognitive neurorehabilitation, it is important to improve the relationship between neuro-sensitized BOLD maps and relevant behavior (Krishnamurthy et al., 2020). Thus the methodological approach of combining CVR maps with BOLD data is extremely important, and from that perspective, systematic experimental and theoretic work from Liau and Liu (2009) have shown that normalization based on division (i.e., voxel-wise BOLD activity divided by corresponding CVR) approach resulted in increased inter-subject variability. On the other hand, group-level normalization by co-variate approach (i.e., voxel-wise covarying out CVR from BOLD activity at group level; Liau and Liu, 2009) retained task sensitivity and improved the predictability of behavior (Krishnamurthy et al., 2020). Thus, the take home message is that mindful selection of BOLD sensitization methodologies is important to accurately interpret treatment-induced changes in rehabilitation.

Delayed and slowed vascular dynamics may have significant implications for the assessment of CVR in the rehabilitation setting. To investigate the temporal characteristics of BOLD CVR measures, conventional CVR analysis methods that are sensitive to magnitude differences in the BOLD response assume that its temporal dynamic properties are consistent across the entire brain, but might not account for delayed responses and slowed dynamics in different tissues or brain areas. Hence, a frequency domain-based transfer function analysis approach might aide in improved identification of regions that present with significantly delayed or slowed responses and provide additional insights into the function of cerebral vessels that are not evident in conventional CVR measures. Such an approach has been proven to enhance the temporal group differences between patients with sickle cell disease and healthy controls (Leung et al., 2016). Also, the dynamic response to a given CO_2_ stimulus may involve transient period of vasodilation or constriction before the blood flow can reach a steady state. Thus advanced analytic approaches (Poublanc et al., 2015) to carefully characterize (A) the dynamic component of CVR that reflects the speed of the cerebrovascular response to hypercapnia, and (B) the static component of CVR that reflects the steady-state reactivity of the vasculature has been shown to be valuable in localizing cerebrovascular compromise in patients with steno-occlusive vasculopathy (Poublanc et al., 2015) and AD (Holmes et al., 2020). In sum, here, we focused on some exciting gasless and analytic approaches of obtaining practical and meaningful CVR maps that are attractive to rehab setting. For more technical aspects of CVR acquisition, types of CO_2_ delivery, etc., such a review can be found elsewhere (Liu et al., 2019).

## Conclusion

The increasing prevalence of cerebrovascular diseases highlights the importance of monitoring cerebrovascular health, as well as a patient’s cognitive and functional changes. Rehabilitation requires an inter-disciplinary care team that relies on biomarkers such as CVR to assist in clinical care decisions, monitor vascular repair, and predict long term recovery in the context of neural plasticity and vascular remodeling (Gabriel-Salazar et al., 2018). Given the inter-subject variability in underlying cerebrovascular physiology and disease profile, there is a strong surge in rehabilitation field to personalize a given treatment (Crosson et al., 2017). From that viewpoint, we have discussed the mechanistic importance of CVR, the diagnostic and prognostic value of CVR, and how it may inform response to specific rehabilitation strategies (exercise and non-invasive brain stimulation, for example). To this end, an accurate measure of treatment-induced change (or no change) using CVR will help uncover the efficacy of a given rehabilitation approach and inform how mechanistic aspects of the rehabilitation implementation (such as dosing, dosing frequency, etc.) can further be optimized to expected outcomes (Crosson et al., 2019).

Importantly, a more comprehensive and holistic understanding of CVR mechanisms and its impairment are extremely important to advance our knowledge in clinical and rehabilitation sciences. From this viewpoint, we have attempted to undertake an integrative physiology approach (that is cerebrovascular control and its integration with other physiological systems such as cardiovascular and pulmonary) to describe the role of CVR, and how understanding the impairment of such mechanisms in various disease models can advance brain rehabilitation programs. Given the versatility of neuroimaging techniques, and that the cerebrovascular control is a complex interplay of various physiological components, it is important to underscore that future work should combine CVR imaging with other neuroimaging approaches that can measure blood flow along with BOLD activity (Krishnamurthy et al., 2020), autoregulation (Whittaker et al., 2019), and brain pH (Ellingson et al., 2019). The future of rehabilitation lies in the search for biomarkers of disease status, progression, and treatment response. It should be noted that although CVR can be used for both diagnostic and prognostic biomarkers, it is likely that the CVR is not sensitive to the same function or underlying mechanism and may have very distinct features and measurement criteria. That is, the CVR maps can be sensitized specifically to underlying mechanisms that are distinct to diagnosis and prediction. Therefore, continued development and testing of CVR-based biomarkers, including accuracy of detection and sensitivity to change, are important factors that could provide continued improvements in rehabilitation strategies.

To conclude, CVR imaging is a valuable tool that has the potential to add significant value to both inpatient acute care and outpatient rehabilitation programs. We also discussed the case for how both CO_2_-based and gasless CVR can find utility in rehabilitation program settings. Finally, multimodal imaging approaches including combining CVR MRI with other modalities to measure pulsatility index, flow mediated dilation, and pulse wave velocity will add further value to provide more accurate and mechanistically driven information for clinical decisions and treatment planning.

## Author Contributions

VK, JS, and JN: conceptualization of the article. VK, JS, LK, JB, AT, IH, and JN: manuscript writing and figures. VK, LK, JS, JB, IH, and JN: manuscript editing. All authors contributed to the article and approved the submitted version.

### Conflict of Interest

The authors declare that the research was conducted in the absence of any commercial or financial relationships that could be construed as a potential conflict of interest.

## References

[ref1] Aaslid R. Lindegaard K. F. Sorteberg W. Nornes H. (1989). Cerebral autoregulation dynamics in humans. Stroke 20, 45–52. 10.1161/01.STR.20.1.45, PMID: 2492126

[ref2] Ainslie P. N. Cotter J. D. George K. P. Lucas S. Murrell C. Shave R.. (2008). Elevation in cerebral blood flow velocity with aerobic fitness throughout healthy human ageing. J. Physiol. 586, 4005–4010. 10.1113/jphysiol.2008.158279, PMID: 18635643 PMC2538930

[ref3] Anstey K. J. Cherbuin N. Budge M. Young J. (2011). Body mass index in midlife and late-life as a risk factor for dementia: a meta-analysis of prospective studies. Obes. Rev. 12, e426–e437. 10.1111/j.1467-789X.2010.00825.x, PMID: 21348917

[ref4] Attems J. Jellinger K. A. (2014). The overlap between vascular disease and Alzheimer’s disease – lessons from pathology. BMC Med. 12:206. 10.1186/s12916-014-0206-2, PMID: 25385447 PMC4226890

[ref5] Bailey D. M. Marley C. J. Brugniaux J. V. Hodson D. New K. J. Ogoh S.. (2013). Elevated aerobic fitness sustained throughout the adult lifespan is associated with improved cerebral hemodynamics. Stroke 44, 3235–3238. 10.1161/STROKEAHA.113.002589, PMID: 23963329

[ref6] Bandettini P. A. Wong E. C. (1997). A hypercapnia-based normalization method for improved spatial localization of human brain activation with fMRI. NMR Biomed. 10, 197–203. 10.1002/(SICI)1099-1492(199706/08)10:4/5<197::AID-NBM466>3.0.CO;2-S, PMID: 9430348

[ref7] Belz G. G. (1995). Elastic properties and Windkessel function of the human aorta. Cardiovasc. Drugs Ther. 9, 73–83. 10.1007/BF00877747, PMID: 7786838

[ref8] Cacciatore F. Abete P. Ferrara N. Paolisso G. Amato L. Canonico S.. (1997). The role of blood pressure in cognitive impairment in an elderly population. Osservatorio Geriatrico Campano group. J. Hypertens. 15, 135–142. 10.1097/00004872-199715020-00003, PMID: 9469788

[ref9] Carr J. Hoiland R. L. Caldwell H. G. Coombs G. B. Howe C. A. Tremblay J. C.. (2020). Internal carotid and brachial artery shear-dependent vasodilator function in young healthy humans. J. Physiol. 598, 5333–5350. 10.1113/JP280369, PMID: 32901919

[ref10] Churchill J. D. Galvez R. Colcombe S. Swain R. A. Kramer A. F. Greenough W. T. (2002). Exercise, experience and the aging brain. Neurobiol. Aging 23, 941–955. 10.1016/S0197-4580(02)00028-3, PMID: 12392797

[ref11] Colcombe S. Kramer A. F. (2003). Fitness effects on the cognitive function of older adults: a meta-analytic study. Psychol. Sci. 14, 125–130. 10.1111/1467-9280.t01-1-01430, PMID: 12661673

[ref12] Cornelissen V. A. Onkelinx S. Goetschalckx K. Thomaes T. Janssens S. Fagard R.. (2014). Exercise-based cardiac rehabilitation improves endothelial function assessed by flow-mediated dilation but not by pulse amplitude tonometry. Eur. J. Prev. Cardiol. 21, 39–48. 10.1177/2047487312460516, PMID: 22962311

[ref13] Coverdale N. S. Badrov M. B. Shoemaker J. K. (2017). Impact of age on cerebrovascular dilation versus reactivity to hypercapnia. J. Cereb. Blood Flow Metab. 37, 344–355. 10.1177/0271678X15626156, PMID: 26759432 PMC5363751

[ref14] Coverdale N. S. Gati J. S. Opalevych O. Perrotta A. Shoemaker J. K. (2014). Cerebral blood flow velocity underestimates cerebral blood flow during modest hypercapnia and hypocapnia. J. Appl. Physiol. 117, 1090–1096. 10.1152/japplphysiol.00285.2014, PMID: 25012027

[ref15] Crosson B. Hampstead B. M. Krishnamurthy L. C. Krishnamurthy V. McGregor K. M. Nocera J. R.. (2017). Advances in neurocognitive rehabilitation research from 1992 to 2017: the Ascension of neural plasticity. Neuropsychology 31, 900–920. 10.1037/neu0000396, PMID: 28857600 PMC5788715

[ref16] Crosson B. Rodriguez A. D. Copland D. Fridriksson J. Krishnamurthy L. C. Meinzer M.. (2019). Neuroplasticity and aphasia treatments: new approaches for an old problem. J. Neurol. Neurosurg. Psychiatry 90, 1147–1155. 10.1136/jnnp-2018-319649, PMID: 31055282 PMC8014302

[ref17] Dai W. Lopez O. L. Carmichael O. T. Becker J. T. Kuller L. H. Gach H. M. (2009). Mild cognitive impairment and alzheimer disease: patterns of altered cerebral blood flow at MR imaging. Radiology 250, 856–866. 10.1148/radiol.2503080751, PMID: 19164119 PMC2680168

[ref18] de Groot J. C. De Leeuw F. E. Oudkerk M. Hofman A. Jolles J. Breteler M. M. (2001). Cerebral white matter lesions and subjective cognitive dysfunction: the Rotterdam Scan Study. Neurology 56, 1539–1545. 10.1212/WNL.56.11.1539, PMID: 11402112

[ref19] Duron E. Hanon O. (2008). Hypertension, cognitive decline and dementia. Arch. Cardiovasc. Dis. 101, 181–189. 10.1016/S1875-2136(08)71801-1, PMID: 18477946

[ref20] Ellingson B. M. Yao J. Raymond C. Chakhoyan A. Khatibi K. Salamon N.. (2019). pH-weighted molecular MRI in human traumatic brain injury (TBI) using amine proton chemical exchange saturation transfer echoplanar imaging (CEST EPI). Neuroimage Clin. 22:101736. 10.1016/j.nicl.2019.101736, PMID: 30826686 PMC6396390

[ref21] Erickson K. I. Kramer A. F. (2009). Aerobic exercise effects on cognitive and neural plasticity in older adults. Br. J. Sports Med. 43, 22–24. 10.1136/bjsm.2008.052498, PMID: 18927158 PMC2853472

[ref22] Esquiva G. Grayston A. Rosell A. (2018). Revascularization and endothelial progenitor cells in stroke. Am. J. Phys. Cell Phys. 315, C664–C674. 10.1152/ajpcell.00200.2018, PMID: 30133323

[ref23] Fan J. L. O’donnell T. Gray C. L. Croft K. Noakes A. K. Koch H.. (2019). Dietary nitrate supplementation enhances cerebrovascular CO_2_ reactivity in a sex-specific manner. J. Appl. Physiol. 127, 760–769. 10.1152/japplphysiol.01116.2018, PMID: 31318615

[ref24] Faraci F. M. Heistad D. D. (1990). Regulation of large cerebral arteries and cerebral microvascular pressure. Circ. Res. 66, 8–17. 10.1161/01.RES.66.1.8, PMID: 2403863

[ref25] Fierstra J. Sobczyk O. Battisti-Charbonney A. Mandell D. M. Poublanc J. Crawley A. P.. (2013). Measuring cerebrovascular reactivity: what stimulus to use? J. Physiol. 591, 5809–5821. 10.1113/jphysiol.2013.259150, PMID: 24081155 PMC3872753

[ref26] Findlay M. D. Dawson J. Dickie D. A. Forbes K. P. McGlynn D. Quinn T.. (2019). Investigating the relationship between cerebral blood flow and cognitive function in hemodialysis patients. J. Am. Soc. Nephrol. 30, 147–158. 10.1681/ASN.2018050462, PMID: 30530658 PMC6317612

[ref27] Fisher J. A. (2016). The CO_2_ stimulus for cerebrovascular reactivity: fixing inspired concentrations vs. targeting end-tidal partial pressures. J. Cereb. Blood Flow Metab. 36, 1004–1011. 10.1177/0271678X16639326, PMID: 27000209 PMC4908627

[ref28] Gabriel-Salazar M. Morancho A. Rodriguez S. Buxo X. Garcia-Rodriguez N. Colell G.. (2018). Importance of Angiogenin and endothelial progenitor cells after rehabilitation both in ischemic stroke patients and in a mouse model of cerebral ischemia. Front. Neurol. 9:508. 10.3389/fneur.2018.00508, PMID: 30008694 PMC6034071

[ref29] Gao Y. Z. Zhang J. J. Liu H. Wu G. Y. Xiong L. Shu M. (2013). Regional cerebral blood flow and cerebrovascular reactivity in Alzheimer’s disease and vascular dementia assessed by arterial spinlabeling magnetic resonance imaging. Curr. Neurovasc. Res. 10, 49–53. 10.2174/156720213804806016, PMID: 23151075

[ref30] Gelinas J. C. Lewis N. C. Harper M. I. Melzer B. Agar G. Rolf J. D.. (2017). Aerobic exercise training does not alter vascular structure and function in chronic obstructive pulmonary disease. Exp. Physiol. 102, 1548–1560. 10.1113/EP086379, PMID: 28857336

[ref31] Geranmayeh F. Wise R. J. Leech R. Murphy K. (2015). Measuring vascular reactivity with breath-holds after stroke: a method to aid interpretation of group-level BOLD signal changes in longitudinal fMRI studies. Hum. Brain Mapp. 36, 1755–1771. 10.1002/hbm.22735, PMID: 25727648 PMC4413362

[ref32] Gerritsen R. J. S. Band G. P. H. (2018). Breath of life: the respiratory vagal stimulation model of contemplative activity. Front. Hum. Neurosci. 12:397. 10.3389/fnhum.2018.00397, PMID: 30356789 PMC6189422

[ref33] Glodzik L. Randall C. Rusinek H. De Leon M. J. (2013). Cerebrovascular reactivity to carbon dioxide in Alzheimer’s disease. J. Alzheimers Dis. 35, 427–440. 10.3233/JAD-122011, PMID: 23478306 PMC3776495

[ref34] Golestani A. M. Wei L. L. Chen J. J. (2016). Quantitative mapping of cerebrovascular reactivity using resting-state BOLD fMRI: validation in healthy adults. NeuroImage 138, 147–163. 10.1016/j.neuroimage.2016.05.025, PMID: 27177763 PMC5148619

[ref35] Grinberg L. T. Heinsen H. (2010). Toward a pathological definition of vascular dementia. J. Neurol. Sci. 299, 136–138. 10.1016/j.jns.2010.08.055, PMID: 20920816 PMC3038202

[ref36] Hadi H. A. Carr C. S. Al Suwaidi J. (2005). Endothelial dysfunction: cardiovascular risk factors, therapy, and outcome. Vasc. Health Risk Manag. 1, 183–198. PMID: 17319104 PMC1993955

[ref37] Hajjar I. Catoe H. Sixta S. Boland R. Johnson D. Hirth V.. (2005). Cross-sectional and longitudinal association between antihypertensive medications and cognitive impairment in an elderly population. J. Gerontol. A Biol. Sci. Med. Sci. 60, 67–73. 10.1093/gerona/60.1.67, PMID: 15741285

[ref38] Hajjar I. Hart M. Chen Y. L. Mack W. Novak V. Chui H. C.. (2013). Antihypertensive therapy and cerebral hemodynamics in executive mild cognitive impairment: results of a pilot randomized clinical trial. J. Am. Geriatr. Soc. 61, 194–201. 10.1111/jgs.12100, PMID: 23350899 PMC3608194

[ref39] Hajjar I. Marmerelis V. Shin D. C. Chui H. (2014). Assessment of cerebrovascular reactivity during resting state breathing and its correlation with cognitive function in hypertension. Cerebrovasc. Dis. 38, 10–16. 10.1159/000365349, PMID: 25171390 PMC4216224

[ref40] Hajjar I. Sorond F. Lipsitz L. A. (2015). Apolipoprotein E, carbon dioxide vasoreactivity, and cognition in older adults: effect of hypertension. J. Am. Geriatr. Soc. 63, 276–281. 10.1111/jgs.13235, PMID: 25688603 PMC4375955

[ref41] Hall C. N. Reynell C. Gesslein B. Hamilton N. B. Mishra A. Sutherland B. A.. (2014). Capillary pericytes regulate cerebral blood flow in health and disease. Nature 508, 55–60. 10.1038/nature13165, PMID: 24670647 PMC3976267

[ref42] Harper A. M. Glass H. I. (1965). Effect of alterations in the arterial carbon dioxide tension on the blood flow through the cerebral cortex at normal and low arterial blood pressures. J. Neurol. Neurosurg. Psychiatry 28, 449–452. 10.1136/jnnp.28.5.449, PMID: 5838479 PMC495935

[ref43] Hoiland R. L. Fisher J. A. Ainslie P. N. (2019). Regulation of the cerebral circulation by arterial carbon dioxide. Compr. Physiol. 9, 1101–1154. 10.1002/cphy.c180021, PMID: 31187899

[ref44] Hoiland R. L. Tymko M. M. Bain A. R. Wildfong K. W. Monteleone B. Ainslie P. N. (2016). Carbon dioxide-mediated vasomotion of extra-cranial cerebral arteries in humans: a role for prostaglandins? J. Physiol. 594, 3463–3481. 10.1113/JP272012, PMID: 26880615 PMC4908020

[ref45] Holmes K. R. Tang-Wai D. Sam K. McKetton L. Poublanc J. Crawley A. P.. (2020). Slowed temporal and parietal cerebrovascular response in patients with Alzheimer’s disease. Can. J. Neurol. Sci. 47, 366–373. 10.1017/cjn.2020.30, PMID: 32051047

[ref46] Holzschneider K. Wolbers T. Roder B. Hotting K. (2012). Cardiovascular fitness modulates brain activation associated with spatial learning. NeuroImage 59, 3003–3014. 10.1016/j.neuroimage.2011.10.021, PMID: 22027496

[ref47] Iadecola C. (2004). Neurovascular regulation in the normal brain and in Alzheimer’s disease. Nat. Rev. Neurosci. 5, 347–360. 10.1038/nrn1387, PMID: 15100718

[ref48] Ikram M. K. De Jong F. J. Van Dijk E. J. Prins N. D. Hofman A. Breteler M. M.. (2006). Retinal vessel diameters and cerebral small vessel disease: the Rotterdam scan study. Brain 129, 182–188. 10.1093/brain/awh688, PMID: 16317022

[ref49] Inaba Y. Chen J. A. Bergmann S. R. (2010). Prediction of future cardiovascular outcomes by flow-mediated vasodilatation of brachial artery: a meta-analysis. Int. J. Card. Imaging 26, 631–640. 10.1007/s10554-010-9616-1, PMID: 20339920

[ref50] In’t Veld B. A. Ruitenberg A. Hofman A. Stricker B. H. Breteler M. M. (2001). Antihypertensive drugs and incidence of dementia: the Rotterdam Study. Neurobiol. Aging 22, 407–412. 10.1016/S0197-4580(00)00241-4, PMID: 11378246

[ref51] Intzandt B. Sabra D. Foster C. Desjardins-Crepeau L. Hoge R. D. Steele C. J.. (2020). Higher cardiovascular fitness level is associated with lower cerebrovascular reactivity and perfusion in healthy older adults. J. Cereb. Blood Flow Metab. 40, 1468–1481. 10.1177/0271678X19862873, PMID: 31342831 PMC7308519

[ref52] Isaacs K. R. Anderson B. J. Alcantara A. A. Black J. E. Greenough W. T. (1992). Exercise and the brain: angiogenesis in the adult rat cerebellum after vigorous physical activity and motor skill learning. J. Cereb. Blood Flow Metab. 12, 110–119. 10.1038/jcbfm.1992.14, PMID: 1370068

[ref53] Ito S. Nagasawa T. Abe M. Mori T. (2009). Strain vessel hypothesis: a viewpoint for linkage of albuminuria and cerebro-cardiovascular risk. Hypertens. Res. 32, 115–121. 10.1038/hr.2008.27, PMID: 19262469

[ref54] Jouett N. P. Watenpaugh D. E. Dunlap M. E. Smith M. L. (2015). Interactive effects of hypoxia, hypercapnia and lung volume on sympathetic nerve activity in humans. Exp. Physiol. 100, 1018–1029. 10.1113/EP085092, PMID: 26132990

[ref55] Junejo R. T. May S. Alsalahi S. Alali M. Ogoh S. Fisher J. P. (2020). Cerebrovascular carbon dioxide reactivity and flow-mediated dilation in young healthy South Asian and Caucasian European men. Am. J. Physiol. Heart Circ. Physiol. 318, H756–H763. 10.1152/ajpheart.00641.2019, PMID: 32083976

[ref56] Khorvash F. Shahnazi H. Saadatnia M. Esteki-Ghashghaei F. (2020). Implementation of home-based health promotion program to improve flow-mediated dilation among patients with subacute stroke. J. Educ. Health Promot. 9:41. 10.4103/jehp.jehp_583_19, PMID: 32318609 PMC7161693

[ref57] Kilander L. Nyman H. Boberg M. Lithell H. (2000). The association between low diastolic blood pressure in middle age and cognitive function in old age. A population-based study. Age Ageing 29, 243–248. 10.1093/ageing/29.3.243, PMID: 10855907

[ref58] Kitazono T. Faraci F. M. Taguchi H. Heistad D. D. (1995). Role of potassium channels in cerebral blood vessels. Stroke 26, 1713–1723. 10.1161/01.STR.26.9.1713, PMID: 7660420

[ref59] Kivipelto M. Helkala E. L. Laakso M. P. Hanninen T. Hallikainen M. Alhainen K.. (2001). Midlife vascular risk factors and Alzheimer’s disease in later life: longitudinal, population based study. BMJ 322, 1447–1451. 10.1136/bmj.322.7300.1447, PMID: 11408299 PMC32306

[ref60] Klein T. Bailey T. G. Wollseiffen P. Schneider S. Askew C. D. (2020). The effect of age on cerebral blood flow responses during repeated and sustained stand to sit transitions. Phys. Rep. 8:e14421. 10.14814/phy2.14421, PMID: 32378357 PMC7202987

[ref61] Kleinloog J. P. D. Mensink R. P. Ivanov D. Adam J. J. Uludag K. Joris P. J. (2019). Aerobic exercise training improves cerebral blood flow and executive function: a randomized, controlled cross-over trial in sedentary older men. Front. Aging Neurosci. 11:333. 10.3389/fnagi.2019.00333, PMID: 31866855 PMC6904365

[ref62] Krainik A. Hund-Georgiadis M. Zysset S. Von Cramon D. Y. (2005). Regional impairment of cerebrovascular reactivity and BOLD signal in adults after stroke. Stroke 36, 1146–1152. 10.1161/01.STR.0000166178.40973.a7, PMID: 15879326

[ref63] Krishnamurthy V. Krishnamurthy L. C. Drucker J. H. Kundu S. Ji B. Hortman K.. (2020). Correcting task fMRI signals for variability in baseline CBF improves BOLD-behavior relationships: a feasibility study in an aging model. Front. Neurosci. 14:336. 10.3389/fnins.2020.00336, PMID: 32425745 PMC7205008

[ref64] Laurent S. Cockcroft J. Van Bortel L. Boutouyrie P. Giannattasio C. Hayoz D.. (2006). Expert consensus document on arterial stiffness: methodological issues and clinical applications. Eur. Heart J. 27, 2588–2605. 10.1093/eurheartj/ehl254, PMID: 17000623

[ref65] Lennox W. G. Gibbs E. L. (1932). The blood flow in the brain and the leg of man, and the changes induced by alteration of blood gases. J. Clin. Invest. 11, 1155–1177. 10.1172/JCI100470, PMID: 16694095 PMC435872

[ref66] Leung J. Duffin J. Fisher J. A. Kassner A. (2016). MRI-based cerebrovascular reactivity using transfer function analysis reveals temporal group differences between patients with sickle cell disease and healthy controls. Neuroimage Clin. 12, 624–630. 10.1016/j.nicl.2016.09.009, PMID: 27722086 PMC5048082

[ref67] Leveille S. G. Kiel D. P. Jones R. N. Roman A. Hannan M. T. Sorond F. A.. (2008). The MOBILIZE Boston Study: design and methods of a prospective cohort study of novel risk factors for falls in an older population. BMC Geriatr. 8:16. 10.1186/1471-2318-8-16, PMID: 18638389 PMC2500010

[ref68] Liau J. Liu T. T. (2009). Inter-subject variability in hypercapnic normalization of the BOLD fMRI response. NeuroImage 45, 420–430. 10.1016/j.neuroimage.2008.11.032, PMID: 19111622 PMC2646818

[ref69] Lindsay J. Laurin D. Verreault R. Hebert R. Helliwell B. Hill G. B.. (2002). Risk factors for Alzheimer’s disease: a prospective analysis from the Canadian Study of Health and Aging. Am. J. Epidemiol. 156, 445–453. 10.1093/aje/kwf074, PMID: 12196314

[ref70] Liu P. De Vis J. B. Lu H. (2019). Cerebrovascular reactivity (CVR) MRI with CO_2_ challenge: a technical review. NeuroImage 187, 104–115. 10.1016/j.neuroimage.2018.03.047, PMID: 29574034 PMC6150860

[ref71] Liu P. Y. Hebrank A. C. Rodrigue K. M. Kennedy K. M. Section J. Park D. C.. (2013). Age-related differences in memory-encoding fMRI responses after accounting for decline in vascular reactivity. NeuroImage 78, 415–425. 10.1016/j.neuroimage.2013.04.053, PMID: 23624491 PMC3694392

[ref72] Liu P. Li Y. Pinho M. Park D. C. Welch B. G. Lu H. (2017). Cerebrovascular reactivity mapping without gas challenges. NeuroImage 146, 320–326. 10.1016/j.neuroimage.2016.11.054, PMID: 27888058 PMC5321860

[ref73] Liu P. Xu C. Lin Z. Sur S. Li Y. Yasar S.. (2020). Cerebrovascular reactivity mapping using intermittent breath modulation. NeuroImage 215:116787. 10.1016/j.neuroimage.2020.116787, PMID: 32278094 PMC7292765

[ref74] Lu H. Xu F. Rodrigue K. M. Kennedy K. M. Cheng Y. Flicker B.. (2011). Alterations in cerebral metabolic rate and blood supply across the adult lifespan. Cereb. Cortex 21, 1426–1434. 10.1093/cercor/bhq224, PMID: 21051551 PMC3097991

[ref75] Maggio P. Salinet A. S. M. Panerai R. B. Robinson T. G. (2013). Does hypercapnia-induced impairment of cerebral autoregulation affect neurovascular coupling? A functional TCD study. J. Appl. Physiol. 115, 491–497. 10.1152/japplphysiol.00327.2013, PMID: 23743398 PMC3742941

[ref76] Manschot S. M. Brands A. M. Van Der Grond J. Kessels R. P. Algra A. Kappelle L. J.. (2006). Brain magnetic resonance imaging correlates of impaired cognition in patients with type 2 diabetes. Diabetes 55, 1106–1113. 10.2337/diabetes.55.04.06.db05-1323, PMID: 16567535

[ref77] Marmarelis V. Z. Shin D. C. Orme M. Rong Z. (2014a). Time-varying modeling of cerebral hemodynamics. IEEE Trans. Biomed. Eng. 61, 694–704. 10.1109/TBME.2013.2287120, PMID: 24184697 PMC4059681

[ref78] Marmarelis V. Z. Shin D. C. Orme M. E. Zhang R. (2014b). Model-based physiomarkers of cerebral hemodynamics in patients with mild cognitive impairment. Med. Eng. Phys. 36, 628–637. 10.1016/j.medengphy.2014.02.025, PMID: 24698010 PMC4076301

[ref79] Marmarelis V. Z. Shin D. C. Tarumi T. Zhang R. (2017). Comparison of model-based indices of cerebral autoregulation and vasomotor reactivity using transcranial Doppler versus near-infrared spectroscopy in patients with amnestic mild cognitive impairment. J. Alzheimers Dis. 56, 89–105. 10.3233/JAD-161004, PMID: 27911329 PMC5240580

[ref80] McAdams-DeMarco M. A. Konel J. Warsame F. Ying H. Fernandez M. G. Carlson M. C.. (2018). Intradialytic cognitive and exercise training may preserve cognitive function. Kidney Int. Rep. 3, 81–88. 10.1016/j.ekir.2017.08.006, PMID: 29340317 PMC5762950

[ref81] McGregor K. M. Nocera J. R. Sudhyadhom A. Patten C. Manini T. M. Kleim J. A.. (2013). Effects of aerobic fitness on aging-related changes of interhemispheric inhibition and motor performance. Front. Aging Neurosci. 5:66. 10.3389/fnagi.2013.00066, PMID: 24198784 PMC3812779

[ref82] McGregor K. M. Zlatar Z. Kleim E. Sudhyadhom A. Bauer A. Phan S.. (2011). Physical activity and neural correlates of aging: a combined TMS/fMRI study. Behav. Brain Res. 222, 158–168. 10.1016/j.bbr.2011.03.042, PMID: 21440574 PMC3713467

[ref83] Miller K. B. Howery A. J. Harvey R. E. Eldridge M. W. Barnes J. N. (2018). Cerebrovascular reactivity and central arterial stiffness in habitually exercising healthy adults. Front. Physiol. 9:1096. 10.3389/fphys.2018.01096, PMID: 30174609 PMC6107836

[ref84] Mitchell G. F. Hwang S. J. Vasan R. S. Larson M. G. Pencina M. J. Hamburg N. M.. (2010). Arterial stiffness and cardiovascular events: the Framingham Heart Study. Circulation 121, 505–511. 10.1161/CIRCULATIONAHA.109.886655, PMID: 20083680 PMC2836717

[ref85] Moraine J. J. Lamotte M. Berre J. Niset G. Leduc A. Naeije R. (1993). Relationship of middle cerebral-artery blood-flow velocity to intensity during dynamic exercise in normal subjects. Eur. J. Appl. Physiol. Occup. Physiol. 67, 35–38. 10.1007/BF00377701, PMID: 8375362

[ref86] Moretti R. Torre P. Antonello R. M. Manganaro D. Vilotti C. Pizzolato G. (2008). Risk factors for vascular dementia: hypotension as a key point. Vasc. Health Risk Manag. 4, 395–402. 10.2147/vhrm.s2434, PMID: 18561514 PMC2496988

[ref87] Morris M. C. Scherr P. A. Hebert L. E. Glynn R. J. Bennett D. A. Evans D. A. (2001). Association of incident Alzheimer disease and blood pressure measured from 13 years before to 2 years after diagnosis in a large community study. Arch. Neurol. 58, 1640–1646. 10.1001/archneur.58.10.1640, PMID: 11594923

[ref88] Murray M. D. Lane K. A. Gao S. Evans R. M. Unverzagt F. W. Hall K. S.. (2002). Preservation of cognitive function with antihypertensive medications: a longitudinal analysis of a community-based sample of African Americans. Arch. Intern. Med. 162, 2090–2096. 10.1001/archinte.162.18.2090, PMID: 12374517

[ref89] Murrell C. J. Cotter J. D. Thomas K. N. Lucas S. J. Williams M. J. Ainslie P. N. (2013). Cerebral blood flow and cerebrovascular reactivity at rest and during sub-maximal exercise: effect of age and 12-week exercise training. Age 35, 905–920. 10.1007/s11357-012-9414-x, PMID: 22669592 PMC3636405

[ref90] Nocera J. Crosson B. Mammino K. McGregor K. M. (2017). Changes in cortical activation patterns in language areas following an aerobic exercise intervention in older adults. Neural Plast. 2017:6340302. 10.1155/2017/6340302, PMID: 28367334 PMC5358467

[ref91] Nocera J. R. McGregor K. M. Hass C. J. Crosson B. (2015). Spin exercise improves semantic fluency in previously sedentary older adults. J. Aging Phys. Act. 23, 90–94. 10.1123/JAPA.2013-0107, PMID: 24425525

[ref92] Novak V. Hajjar I. (2010). The relationship between blood pressure and cognitive function. Nat. Rev. Cardiol. 7, 686–698. 10.1038/nrcardio.2010.161, PMID: 20978471 PMC3328310

[ref93] O’Rourke M. F. Safar M. E. (2005). Relationship between aortic stiffening and microvascular disease in brain and kidney: cause and logic of therapy. Hypertension 46, 200–204. 10.1161/01.HYP.0000168052.00426.65, PMID: 15911742

[ref94] Ozturk E. D. Tan C. O. (2018). Human cerebrovascular function in health and disease: insights from integrative approaches. J. Physiol. Anthropol. 37:4. 10.1186/s40101-018-0164-z, PMID: 29454381 PMC5816507

[ref95] Palazzo P. Maggio P. Passarelli F. Altavilla R. Altamura C. Pasqualetti P.. (2013). Lack of correlation between cerebral vasomotor reactivity and flow-mediated dilation in subjects without vascular disease. Ultrasound Med. Biol. 39, 10–15. 10.1016/j.ultrasmedbio.2012.08.022, PMID: 23141904

[ref96] Park Y. J. Park Y. B. (2012). Clinical utility of paced breathing as a concentration meditation practice. Complement. Ther. Med. 20, 393–399. 10.1016/j.ctim.2012.07.008, PMID: 23131369

[ref97] Perry B. G. Lucas S. J. Thomas K. N. Cochrane D. J. Mundel T. (2014). The effect of hypercapnia on static cerebral autoregulation. Phys. Rep. 2:e12059. 10.14814/phy2.12059, PMID: 24973333 PMC4208638

[ref98] Poublanc J. Crawley A. P. Sobczyk O. Montandon G. Sam K. Mandell D. M.. (2015). Measuring cerebrovascular reactivity: the dynamic response to a step hypercapnic stimulus. J. Cereb. Blood Flow Metab. 35, 1746–1756. 10.1038/jcbfm.2015.114, PMID: 26126862 PMC4635229

[ref99] Prakash R. S. Patterson B. Janssen A. Abduljalil A. Boster A. (2011). Physical activity associated with increased resting-state functional connectivity in multiple sclerosis. J. Int. Neuropsychol. Soc. 17, 986–997. 10.1017/S1355617711001093, PMID: 22040897

[ref100] Qiu C. Von Strauss E. Fastbom J. Winblad B. Fratiglioni L. (2003). Low blood pressure and risk of dementia in the Kungsholmen project: a 6-year follow-up study. Arch. Neurol. 60, 223–228. 10.1001/archneur.60.2.223, PMID: 12580707

[ref101] Qiu C. Winblad B. Fratiglioni L. (2005). The age-dependent relation of blood pressure to cognitive function and dementia. Lancet Neurol. 4, 487–499. 10.1016/S1474-4422(05)70141-1, PMID: 16033691

[ref102] Qiu C. Winblad B. Fratiglioni L. (2009). Low diastolic pressure and risk of dementia in very old people: a longitudinal study. Dement. Geriatr. Cogn. Disord. 28, 213–219. 10.1159/000236913, PMID: 19752556

[ref103] Rajendran P. Rengarajan T. Thangavel J. Nishigaki Y. Sakthisekaran D. Sethi G.. (2013). The vascular endothelium and human diseases. Int. J. Biol. Sci. 9, 1057–1069. 10.7150/ijbs.7502, PMID: 24250251 PMC3831119

[ref104] Regan R. E. Fisher J. A. Duffin J. (2014). Factors affecting the determination of cerebrovascular reactivity. Brain and Behav. 4, 775–788. 10.1002/brb3.275, PMID: 25328852 PMC4188369

[ref105] Richiardi J. Monsch A. U. Haas T. Barkhof F. Van De Ville D. Radu E. W.. (2015). Altered cerebrovascular reactivity velocity in mild cognitive impairment and Alzheimer’s disease. Neurobiol. Aging 36, 33–41. 10.1016/j.neurobiolaging.2014.07.020, PMID: 25146454

[ref106] Rose K. M. Couper D. Eigenbrodt M. L. Mosley T. H. Sharrett A. R. Gottesman R. F. (2010). Orthostatic hypotension and cognitive function: the Aherosclerosis Risk in Communities Study. Neuroepidemiology 34, 1–7. 10.1159/000255459, PMID: 19893322 PMC2857621

[ref107] Sander K. Hof U. Poppert H. Conrad B. Sander D. (2005). Improved cerebral vasoreactivity after statin administration in healthy adults. J. Neuroimaging 15, 266–270. 10.1111/j.1552-6569.2005.tb00320.x, PMID: 15951410

[ref108] Santos-Parker J. R. Larocca T. J. Seals D. R. (2014). Aerobic exercise and other healthy lifestyle factors that influence vascular aging. Adv. Physiol. Educ. 38, 296–307. 10.1152/advan.00088.2014, PMID: 25434012 PMC4315444

[ref109] Savva G. M. Stephan B. C. M. Alzheimer’s Society Vascular Dementia Systematic Review Group (2010). Epidemiological studies of the effect of stroke on incident dementia: a systematic review. Stroke 41, e41–e46. 10.1161/STROKEAHA.109.559880, PMID: 19910553

[ref110] Seals D. R. Kaplon R. E. Gioscia-Ryan R. A. Larocca T. J. (2014). You’re only as old as your arteries: translational strategies for preserving vascular endothelial function with aging. Physiology 29, 250–264. 10.1152/physiol.00059.2013, PMID: 24985329 PMC4103060

[ref111] Sharp S. I. Aarsland D. Day S. Sonnesyn H. Alzheimer’s Society Vascular Dementia Systematic Review Group Ballard C. (2011). Hypertension is a potential risk factor for vascular dementia: systematic review. Int. J. Geriatr. Psychiatry 26, 661–669. 10.1002/gps.2572, PMID: 21495075

[ref112] Shekhar S. Liu R. Travis O. K. Roman R. J. Fan F. (2017). Cerebral autoregulation in hypertension and ischemic stroke: a mini review. J. Pharm. Sci. Exp. Pharmacol. 2017, 21–27. PMID: 29333537 PMC5765762

[ref113] Sierra C. Domenech M. Camafort M. Coca A. (2012). Hypertension and mild cognitive impairment. Curr. Hypertens. Rep. 14, 548–555. 10.1007/s11906-012-0315-2, PMID: 23073614

[ref114] Skolarus L. E. Freedman V. A. Feng C. Wing J. J. Burke J. F. (2016). Care received by elderly US stroke survivors may be underestimated. Stroke 47, 2090–2095. 10.1161/STROKEAHA.116.012704, PMID: 27387990 PMC4961527

[ref115] Smith J. C. Nielson K. A. Woodard J. L. Seidenberg M. Durgerian S. Antuono P.. (2011). Interactive effects of physical activity and APOE-epsilon4 on BOLD semantic memory activation in healthy elders. NeuroImage 54, 635–644. 10.1016/j.neuroimage.2010.07.070, PMID: 20691792 PMC2962671

[ref116] Sobczyk O. Battisti-Charbonney A. Poublanc J. Crawley A. P. Sam K. Fierstra J.. (2015). Assessing cerebrovascular reactivity abnormality by comparison to a reference atlas. J. Cereb. Blood Flow Metab. 35, 213–220. 10.1038/jcbfm.2014.184, PMID: 25388679 PMC4426737

[ref117] Sobczyk O. Crawley A. P. Poublanc J. Sam K. Mandell D. M. Mikulis D. J.. (2016). Identifying significant changes in cerebrovascular reactivity to carbon dioxide. AJNR Am. J. Neuroradiol. 37, 818–824. 10.3174/ajnr.A4679, PMID: 26846924 PMC7960311

[ref118] Sperling R. Mormino E. Johnson K. (2014). The evolution of preclinical Alzheimer’s disease: implications for prevention trials. Neuron 84, 608–622. 10.1016/j.neuron.2014.10.038, PMID: 25442939 PMC4285623

[ref119] Sprick J. D. Nocera J. R. Hajjar I. O’neill W. C. Bailey J. Park J. (2020). Cerebral blood flow regulation in end-stage kidney disease. Am. J. Physiol. Ren. Physiol. 319, F782–F791. 10.1152/ajprenal.00438.2020, PMID: 32985235 PMC7789989

[ref120] Szabo K. Lako E. Juhasz T. Rosengarten B. Csiba L. Olah L. (2011). Hypocapnia induced vasoconstriction significantly inhibits the neurovascular coupling in humans. J. Neurol. Sci. 309, 58–62. 10.1016/j.jns.2011.07.026, PMID: 21831399

[ref121] Tanabe T. Maeda S. Miyauchi T. Iemitsu M. Takanashi M. Irukayama-Tomobe Y.. (2003). Exercise training improves ageing-induced decrease in eNOS expression of the aorta. Acta Physiol. Scand. 178, 3–10. 10.1046/j.1365-201X.2003.01100.x, PMID: 12713509

[ref122] Thomas B. P. Yezhuvath U. S. Tseng B. Y. Liu P. Levine B. D. Zhang R.. (2013). Life-long aerobic exercise preserved baseline cerebral blood flow but reduced vascular reactivity to CO_2_. J. Magn. Reson. Imaging 38, 1177–1183. 10.1002/jmri.24090, PMID: 23526811 PMC3695025

[ref123] Toledo J. B. Arnold S. E. Raible K. Brettschneider J. Xie S. X. Grossman M.. (2013). Contribution of cerebrovascular disease in autopsy confirmed neurodegenerative disease cases in the National Alzheimer’s Coordinating Centre. Brain 136, 2697–2706. 10.1093/brain/awt188, PMID: 23842566 PMC3858112

[ref124] Tsao C. W. Himali J. J. Beiser A. S. Larson M. G. Decarli C. Vasan R. S.. (2016). Association of arterial stiffness with progression of subclinical brain and cognitive disease. Neurology 86, 619–626. 10.1212/WNL.0000000000002368, PMID: 26791155 PMC4762417

[ref125] Vaynman S. Gomez-Pinilla F. (2006). Revenge of the “sit”: how lifestyle impacts neuronal and cognitive health through molecular systems that interface energy metabolism with neuronal plasticity. J. Neurosci. Res. 84, 699–715. 10.1002/jnr.20979, PMID: 16862541

[ref126] Verbree J. Bronzwaer A. S. Ghariq E. Versluis M. J. Daemen M. J. Van Buchem M. A.. (2014). Assessment of middle cerebral artery diameter during hypocapnia and hypercapnia in humans using ultra-high-field MRI. J. Appl. Physiol. 117, 1084–1089. 10.1152/japplphysiol.00651.2014, PMID: 25190741

[ref127] Vermeer S. E. Prins N. D. Den Heijer T. Hofman A. Koudstaal P. J. Breteler M. M. (2003). Silent brain infarcts and the risk of dementia and cognitive decline. N. Engl. J. Med. 348, 1215–1222. 10.1056/NEJMoa022066, PMID: 12660385

[ref128] Waldstein S. R. Giggey P. P. Thayer J. F. Zonderman A. B. (2005). Nonlinear relations of blood pressure to cognitive function: the Baltimore Longitudinal Study of Aging. Hypertension 45, 374–379. 10.1161/01.HYP.0000156744.44218.74, PMID: 15699446

[ref129] Waldstein S. R. Rice S. C. Thayer J. F. Najjar S. S. Scuteri A. Zonderman A. B. (2008). Pulse pressure and pulse wave velocity are related to cognitive decline in the Baltimore Longitudinal Study of Aging. Hypertension 51, 99–104. 10.1161/HYPERTENSIONAHA.107.093674, PMID: 18025297

[ref130] Walker A. E. Eskurza I. Pierce G. L. Gates P. E. Seals D. R. (2009). Modulation of vascular endothelial function by low-density lipoprotein cholesterol with aging: influence of habitual exercise. Am. J. Hypertens. 22, 250–256. 10.1038/ajh.2008.353, PMID: 19114985 PMC2921324

[ref131] Walker A. E. Kaplon R. E. Pierce G. L. Nowlan M. J. Seals D. R. (2014). Prevention of age-related endothelial dysfunction by habitual aerobic exercise in healthy humans: possible role of nuclear factor kappaB. Clin. Sci. 127, 645–654. 10.1042/CS20140030, PMID: 24947434 PMC4408779

[ref132] Wasylyshyn C. Verhaeghen P. Sliwinski M. J. (2011). Aging and task switching: a meta-analysis. Psychol. Aging 26, 15–20. 10.1037/a0020912, PMID: 21261411 PMC4374429

[ref133] Wharton W. Goldstein F. C. Zhao L. Steenland K. Levey A. I. Hajjar I. (2015). Modulation of renin-angiotensin system may slow conversion from mild cognitive impairment to Alzheimer’s disease. J. Am. Geriatr. Soc. 63, 1749–1756. 10.1111/jgs.13627, PMID: 26389987 PMC4743657

[ref134] Whitmer R. A. Sidney S. Selby J. Johnston S. C. Yaffe K. (2005). Midlife cardiovascular risk factors and risk of dementia in late life. Neurology 64, 277–281. 10.1212/01.WNL.0000149519.47454.F2, PMID: 15668425

[ref135] Whittaker J. R. Driver I. D. Venzi M. Bright M. G. Murphy K. (2019). Cerebral autoregulation evidenced by synchronized low frequency oscillations in blood pressure and resting-state fMRI. Front. Neurosci. 13:433. 10.3389/fnins.2019.00433, PMID: 31133780 PMC6514145

[ref136] Willie C. K. MacLeod D. B. Shaw A. D. Smith K. J. Tzeng Y. C. Eves N. D.. (2012). Regional brain blood flow in man during acute changes in arterial blood gases. J. Physiol. 590, 3261–3275. 10.1113/jphysiol.2012.228551, PMID: 22495584 PMC3459041

[ref137] Willie C. K. Tzeng Y. C. Fisher J. A. Ainslie P. N. (2014). Integrative regulation of human brain blood flow. J. Physiol. 592, 841–859. 10.1113/jphysiol.2013.268953, PMID: 24396059 PMC3948549

[ref138] Yamada M. Huang Z. Dalkara T. Endres M. Laufs U. Waeber C.. (2000). Endothelial nitric oxide synthase-dependent cerebral blood flow augmentation by L-arginine after chronic statin treatment. J. Cereb. Blood Flow Metab. 20, 709–717. 10.1097/00004647-200004000-00008, PMID: 10779015

[ref139] Yasar S. Corrada M. Brookmeyer R. Kawas C. (2005). Calcium channel blockers and risk of AD: the Baltimore Longitudinal Study of Aging. Neurobiol. Aging 26, 157–163. 10.1016/j.neurobiolaging.2004.03.009, PMID: 15582745

[ref140] Zhao P. Alsop D. C. Abduljalil A. Selim M. Lipsitz L. Novak P.. (2009). Vasoreactivity and peri-infarct hyperintensities in stroke. Neurology 72, 643–649. 10.1212/01.wnl.0000342473.65373.80, PMID: 19221298 PMC2677535

[ref141] Zhao Z. Q. Qin L. Reid B. Pu J. Hara T. Zhao M. (2012). Directing migration of endothelial progenitor cells with applied DC electric fields. Stem Cell Res. 8, 38–48. 10.1016/j.scr.2011.08.001, PMID: 22099019 PMC3238468

[ref142] Zieman S. J. Melenovsky V. Kass D. A. (2005). Mechanisms, pathophysiology, and therapy of arterial stiffness. Arterioscler. Thromb. Vasc. Biol. 25, 932–943. 10.1161/01.ATV.0000160548.78317.29, PMID: 15731494

[ref143] Zimmerman B. Sutton B. P. Low K. A. Fletcher M. A. Tan C. H. Schneider-Garces N.. (2014). Cardiorespiratory fitness mediates the effects of aging on cerebral blood flow. Front. Aging Neurosci. 6:59. 10.3389/fnagi.2014.00059, PMID: 24778617 PMC3985032

